# Pore Structure Tuning of Poly-EGDMA Biomedical Material by Varying the O-Quinone Photoinitiator

**DOI:** 10.3390/polym15112558

**Published:** 2023-06-02

**Authors:** Vladimir V. Yudin, Margarita P. Shurygina, Marfa N. Egorikhina, Diana Ya. Aleynik, Daria D. Linkova, Irina N. Charykova, Roman S. Kovylin, Sergey A. Chesnokov

**Affiliations:** 1 G. A. Razuvaev Institute of Organometallic Chemistry of the Russian Academy of Sciences, 49 Tropinina, 603950 Nizhny Novgorod, Russia; yudin@iomc.ras.ru (V.V.Y.); shurygina_rina@mail.ru (M.P.S.); sch@iomc.ras (S.A.C.); 2 Federal State Budgetary Educational Institution of Higher Education, Privolzhsky Research Medical University of the Ministry of Health of the Russian Federation, 10/1 Ploshchad Minina i Pozharskogo, 603005 Nizhny Novgorod, Russia; egorihina.marfa@yandex.ru (M.N.E.); daleynik@yandex.ru (D.Y.A.); linckovadaria@yandex.ru (D.D.L.); irina-ch0709@yandex.ru (I.N.C.)

**Keywords:** photoinitiator, phenanthrenequinone, camphorquinone, o-benzoquinone, ethylene glycol dimethacrylate, visible radiation, porosity, strength properties, scanning electron microscopy, cytotoxicity

## Abstract

Porous polymer monoliths with thicknesses of 2 and 4 mm were obtained via polymerization of ethylene glycol dimethacrylate (EGDMA) under the influence visible-light irradiation in the presence of a 70 wt% 1-butanol porogenic agent and o-quinone photoinitiators. The o-quinones used were: 3,5-di-tret-butyl-benzoquinone-1,2 (35Q), 3,6-di-tret-butyl-benzoquinone-1,2 (36Q), camphorquinone (CQ), and 9,10-phenanthrenequinone (PQ). Porous monoliths were also synthesized from the same mixture but using 2,2′-azo-bis(iso-butyronitrile) (AIBN) at 100 °C instead o-quinones. According to the results of scanning electron microscopy, all the resulting samples were conglomerates of spherical, polymeric particles with pores between them. Use of mercury porometry showed that the interconnected pore systems of all the polymers were open. The average pore size, *D_mod_*, in such polymers strongly depended on both the nature of the initiator and the method of initiation of polymerization. For polymers obtained in the presence of AIBN, the *D_mod_* value was as low as 0.8 μm. For polymers obtained via photoinitiation in the presence of 36Q, 35Q, CQ, and PQ, the *D_mod_* values were significantly greater, i.e., 9.9, 6.4, 3.6, and 3.7 μm, respectively. The compressive strength and Young’s modulus of the porous monoliths increased symbatically in the series PQ < CQ < 36Q < 35Q < AIBN with decreasing proportions of large pores (over 12 μm) in their polymer structures. The photopolymerization rate of the EGDMA and 1-butanol, 30:70 wt% mixture was maximal for PQ and minimal for 35Q. All polymers tested were non-cytotoxic. Based on the data from MTT testing, it can be noted that the polymers obtained via photoinitiation were characterized by their positive effect on the proliferative activity of human dermal fibroblasts. This makes them promising osteoplastic materials for clinical trials.

## 1. Introduction

The speed of development of regenerative medicine requires increasingly innovative materials with properties similar to body tissues. A lot of research is currently underway in the field of synthesis and modification [1] aimed both at creating new biomaterials and improving aspects of regeneration [2]. Various combinations of approaches and materials are used to address the challenges of regenerative medicine and of cell death, as well as to improve vascularization and tissue regeneration [3,4]. Structures for replacing bone tissue defects have become some of the materials in greatest demand because of the rising extent of traumatic bone lesions [5,6], osteoporosis [7,8,9], and malignant diseases of bone [10]. The most important characteristic of such scaffold materials, used for providing effective restoration of bone defects, is that their porous structure largely determines the properties of the final product. Thus, the scaffold properties are influenced by various parameters, the main ones being pore size distribution and pore volumes and shapes, along with their interconnections. The interconnected open porosity with sufficient pore size, allowing for fluid circulation and nutrient exchange, chemotaxis, cell adhesion, proliferation and differentiation, and, subsequently, vascularization and new tissue formation, contributes to the successful use of such scaffold frameworks for bone tissue engineering [11,12]. The approaches to creating these materials are numerous [13]. One of the options for obtaining porous materials is polymerization of compositions consisting of monomers and pore-forming components. Due to thermodynamic incompatibility between the formed polymer and the porogen, microsyneresis occurs during polymerization. As a result, pores filled with porogen form within the body of the polymer [14]. Irradiation with UV or visible light is an effective method for initiating polymerization when implementing this approach. Photoinitiation allows polymerization across a wide temperature range and, therefore, it becomes possible to use low-boiling-point porogenic components that are not suitable for thermopolymerizing compositions. Pore formation during polymerization is accompanied by clouding of the polymerizing medium. Due to absorption and scattering of the initiating radiation, its intensity decreases when passing through the polymerizing layer, and this leads to the photopolymerization process slowing down and ceasing. Most photopolymerizing compositions (PPCs) are sensitive to UV radiation, which is effectively scattered by inhomogeneities of the medium but is absorbed by the composition components. Therefore, UV-initiated photopolymerization only allows for the formation of thin, porous polymer layers with thicknesses (h) of less than a millimeter. However, many biomedical applications require bulkier and thicker porous structures. Accordingly, new methods need to be developed to generate more substantial structures with thicknesses of up to several millimeters. The transition from UV initiation to visible-light initiation enables the thickness of the photocured layer to be increased. Previously, we had obtained *h* = 4 mm porous polymer monoliths via visible-light photopolymerization of compositions based on dimethacrylic monomers [15]. The influence of the nature and concentration of the monomers and pore-forming agents on the structure and properties of the porous polymers has also been studied. The polymers showed zero cytotoxicity and good adhesive properties, and they enabled high proliferative activity of mesenchymal stem cells (MSCs) on the surface of the samples [16,17,18]. Obviously, apart from the selection of monomers and pore-forming agents [19], the nature of the photoinitiator and its concentration should have a significant influence on the properties of the resulting porous polymers [20,21]. These are related to the photopolymerization rate [22], which can be set by both the nature of the photoinitiator and the intensity of the actinic radiation. The transition to systems sensitive to visible radiation is usually associated with the use of quinone photoinitiators [23,24,25,26,27]. These are type II photoinitiators. On the other hand, it is well known that quinones are inhibitors of radical polymerization [28,29,30]. This dualism of quinone behavior in photopolymerizing media predetermines the nature of the influence of the quinones not only on the kinetics of the polymerization process, but also on the pore characteristics of the resulting porous polymer. To date, there have been no investigations on the influence of the initiator structure on the formed polymers’ pore characteristics for radical photopolymerization under the action of visible light.

**Scheme 1:**
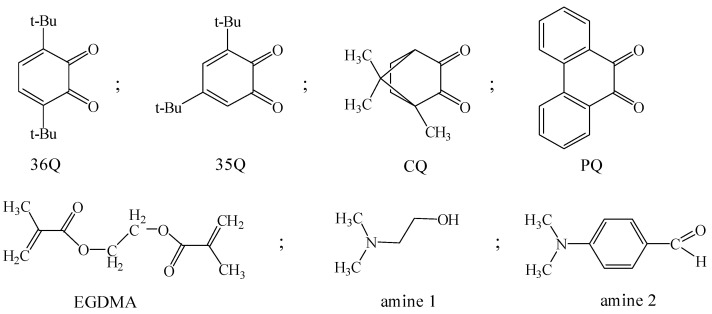
The structure of dimethacrylate and components of the photoinitiating system (quinones and amines).

The present paper deals with the properties of porous polymers obtained via visible-light photopolymerization using photoinitiating systems based on four different quinone photoinitiators: 3,6-di-*tret*-butyl-benzoquinone-1,2 (36Q), 3,5-di-*tret*-butyl-benzoquinone-1,2 (35Q), 9,10-phenanthrenequinone (PQ), and camphorquinone (CQ). The following factors determined our choice of o-quinones. Quinone 36Q effectively initiates photopolymerization of dimethacrylates even in thick layers [31] and is a weak inhibitor [23,32,33,34], as shown by the example of methyl methacrylate (MMA) polymerization initiated by azobisisobutyronitrile (AIBN). Quinone 35Q is also a photoinitiator of radical polymerization, but it has a much greater inhibitory capacity than 36Q [23,33,34,35]. Camphorquinone is one of the best known and most used photopolymerization initiators [36,37,38,39,40,41,42,43], but it is less effective than 36Q in photocuring thick layers [23]. All three listed photoinitiators are useless without the addition of co-initiators, usually tertiary amines [24,44,45]. In contrast, PQ requires no co-initiator to photoinitiate polymerization [43,46,47]. A mixture of ethylene glycol dimethacrylate (EGDMA) and 1-butanol in a 30:70 mass ratio was used for photopolymerization, leading to the formation of porous polymers; this was the approach previously used to obtain biocompatible polymers in a 36Q photoinitiating system [16]. The structural formulas of the studied quinones, EGDMA, and amines are shown in Scheme 1.

This article reports, for the first time, on the relationship between the kinetics of photopolymerization, pore characteristics, morphology, and the strength properties of porous polymers synthesized using visible-light photopolymerization from an EGDMA and 1-butanol mixture with initiator systems based on the listed o-quinones. For comparison, porous polymers obtained via polymerization of the same reaction mixture, but using 2,2′-azo-bis(iso-butyronitrile) (AIBN) at T = 100 °C as the initiator, were also studied. To assess the possibility of further application of the resulting materials in biomedical applications, the effect of the photoinitiating systems on the cytotoxicity of these polymers was additionally investigated.

## 2. Materials and Methods

*Materials.* Ethylene glycol dimethacrylate (EGDMA) (98%, Aldrich, St. Louis, MO, USA); 1-butanol (BuOH) (99.5%, Aldrich, St. Louis, MO, USA); dimethoxyethane (DME) (Aldrich, St. Louis, MO, USA); dimethyl sulfoxide (DMSO) (Aldrich, St. Louis, MO, USA); 9,10-phenanthrenequinone (Fluka, Buchs, Switzerland); camphoroquinone (Aldrich, St. Louis, MO, USA); 3,5-di-*tret*-butyl-benzoquinone-1,2 (Aldrich, St. Louis, MO, USA); 3,6-di-*tret*-butyl-benzoquinone-1,2 (Aldrich, St. Louis, MO, USA); 3-[4,5-dimethylthiazol-2-yl]-2,5-diphenyltetrazolium bromide (MTT) (Aldrich, St. Louis, MO, USA); N,N-dimethyl ethanolamine (Acros, Morris Plains, NJ, USA); and n-(N,N-dimethylamino)benzaldehyde (Aldrich, St. Louis, MO, USA) were each used without further purification. 2,2′-azo-bis(iso-butyronitrile) (Reahim, Moscow, Russia) was purified via double recrystallization from methanol. Eagle’s medium modified using Dulbecco (DMEM) (Aldrich, St. Louis, MO, USA) and calf fetal serum (PanEco LLC., Moscow, Russia) were also used.

*Synthesis of porous monoliths.* Porous polymers were obtained according to an established procedure [15]. The PPCs were prepared by dissolving 0.1 wt% o-quinone in an EGDMA and 1-butanol mixture with a 30:70 weight ratio at T = 25 °C. When 35Q and 36Q were used, 1 wt% N,N-dimethylethanolamine (amine1) was added to the solution; when CQ was used, 0.5 wt% n-(N,N-dimethylamino)benzaldehyde (amine2) was added to the solution. The prepared mixture was poured into a mold consisting of two flat silicate glasses with either a spacer of 2 or 4 mm thickness. The irradiation was started one minute after pouring the composition into the mold. A Philips UHP halogen lamp (400–750 nm, 190 W) was used to initiate polymerization. The composition was irradiated for 2 h with illuminance *I* = 50 kLx.

As an alternative option to obtaining a porous monolith via thermopolymerization, a reaction mixture was prepared by dissolving 0.1 wt% AIBN in an EGDMA and 1-butanol mixture, 30:70 wt% at T = 25 °C. The finished composition was poured into a mold consisting of two flat silicate glasses with either a 2 or 4 mm spacer. The mold was placed in a thermal oven and heated at T = 100 °C for 60 min.

Upon completion of polymerization, the porous poly-EGDMA monoliths were removed from the molds and washed with isopropyl alcohol in a Soxhlet apparatus at least 50 times (about 8 h). The polymer monoliths were vacuumized at 60 °C for 24 h to remove the alcohol.

*Characterization.* The structural characteristics of the resulting porous polymer monoliths (mean pore size, *D_mod_*; specific surface, *S_sp_*; porosity as skeletal density, *ε_sk_*; and porosity as mercury intrusion, *ε_Hg_*) were determined via mercury porometry using a PASCAL EVO 140/440 (Thermo Scientific, Rodano, Italy). Skeletal density is defined as the weight of a dry sample referred to the “skeleton” volume of the sample, which is the volume of the sample excluding all the open pores. Mercury intrusion density is defined as the weight of the dry sample referred to the volume of the sample, excluding any pores that may be filled with mercury. Pre-cut samples of the porous polymers weighing 0.2–0.5 g were kept in a desiccator for at least three hours at 110 °C. Then the samples were placed in a glass dilatometer and analyzed. The pore size was determined to be in the 3.6 nm to 120 μm range, and this corresponded to the 10 Pa to 400 MPa pressure applied to the sample. The surfaces of the porous polymer monoliths were examined via scanning electron microscopy (SEM) using a Regulus SU8100 (Hitachi, Tokyo, Japan) microscope. The sizes of both the pores and polymer globules were estimated from the SEM images. The strength characteristics, Young’s modulus at compression (*E*) and destructive stress at compression (*σ*), were studied according to ISO 604:2002 [48]. Each porous poly-EGDMA monolith was cut into 10 × 15 × 4 mm blocks using a blade. Compression tests were run on an Autograph AGX-V 5 kN (Shimadzu, Tokyo, Japan) universal testing machine at a 5 mm/min strain rate using the 10 × 15 × 4 mm samples. The compression modulus was calculated from the slope of the linear area. The presented *E* and *σ* data were averaged over six poly-EGDMA samples for each composition.

*Kinetics of photopolymerization.* The kinetics of the photopolymerization of the EGDMA and 1-butanol mixture were studied via infrared spectrophotometry using a Fourier-spectrometer, FT-801 (Simex, Novosibirsk, Russia) and an NPBO-A unit with a diamond element in the 400 to 1700 cm^−1^ range with 4 cm^−1^ resolution using the method of broken total internal refraction. The photopolymerizing composition was prepared by dissolving 0.1 wt% of the o-quinones in a 30:70 wt% EGDMA and 1-butanol mixture at T = 25 °C in air. A N,N-dimethylethanolamine (1 wt%) co-initiator was added to the 36Q and 35Q solutions, while p-(N,N-dimethylamino)benzaldehyde (5-fold molar excess) was added to the CQ solution as its co-initiator. Each prepared composition was poured into a 2 mm-high ABS plastic limiting ring fixed to the IR spectrometer recording surface; the composition was covered with silicate glass and irradiated with the full light flux of a Philips UHP lamp (400–750 nm, 190 W). The illuminance level in the registration area was 50 kLx. The monomer-to-PPC conversion was determined using the ratio between the intensities of variation of the absorption band of the monomer C=C bond (*v* = 1636 cm^−1^) and the CH_3_ absorption band of the methacrylate fragment group (*v* = 1453 cm^−1^). From the data obtained, the dependences between the monomer conversion and PPC irradiation times, the limit conversion *P_lim_*, and the induction period *T_ind_* were plotted. The maximum photopolymerization rate, *W_max_*, for each PPC was determined using the tangent of the greatest angle of slope to the kinetic curve. The values quoted were determined by results from at least three experiments with a reproducibility of results within 10%. The intensity of the initiating radiation was monitored using a combined Luxmeter + UV-radiometer, the TKA-PKM 06 (NTP TKA LLC., St. Petersburg, Russia).

*Photoreduction kinetics of o-benzoquinones.* The photoreduction kinetics of o-benzoquinones were studied spectrophotometrically via the loss of the absorption band representing the o-quinone using the well-known method [49]. A solution containing 6 × 10^−4^ M o-quinone and N,N-dimethylethanolamine (1:5 quinone-amine molar ratio) in a 30:70 wt% mixture of DME and BuOH was placed in a spectrophotometric cuvette with a thickness of 1 cm and exposed at 30 cm from the focusing device. The solution was irradiated with the full light flux of a Philips UHP lamp (Philips, Eindhoven, The Netherlands, 400–750 nm, 190 W). Illuminance in the registration area was determined using a TKA-PKM 06 (NTP TKA LLC, St. Petersburg, Russia) luxmeter and was 50 kLx. Electronic absorption spectra were recorded on an SF-56 spectrometer (LOMO, St. Petersburg, Russia). The observed quinone photoreduction rate constant, *k_H_*, was determined graphically from the slope tangent of the straight-line plot ln ([*A*_0_]/[*A_t_*]) − *t*. The numerical value of the effective rate constant was averaged over three measurements with a reproducibility of results within 10%.

*Determination of cytotoxicity.* To assess the biological characteristics of the porous polymer monoliths and the possibility of their subsequent application in biomedicine, the levels of cytotoxicity of the samples were evaluated using a standard MTT assay [50]. This method is based on living cells’ ability to reduce yellow bromide 3-(4,5-dimethylthiazol-2-yl)-2,5-tetrazolium (MTT) into purple intracellular MTT-formazan crystals, soluble in isopropanol or dimethyl sulfoxide (DMSO). The amount of reduced product was measured photometrically at 540 nm. A statistically significant decrease in the optical density of the experimental samples compared to control samples as recorded on a tablet reader would allow us to conclude that the tested substance has a cytotoxic effect on the cells.

The MTT method is an extraction method. Samples of 500 mg each were taken to obtain extracts. To each sample, 5 mL of nutrient medium were added, and the samples were incubated for 1 day or 7 days to obtain the extracts. After incubation, the extracts were decanted from the samples and analyzed. Characterized cultures of human skin fibroblasts of passages 4–6 were used as test cultures. Cell cultures were obtained from the biotechnology laboratory of the Federal State Budgetary Educational Institution of Higher Education PIMU of the Ministry of Health of Russia. All test cultures were sterile, not contaminated with mycoplasmas or viruses. The cell viability before introduction into the study was 97–98%. The cells of the cultures were morphologically homogeneous, fusiform, or star-shaped with clear contours and pronounced processes. The phenotypes of the cells corresponded to those of mesenchymal cells: CD 90+, CD 105+, CD 73+, CD10+, CD45−, CD 14−, CD 34−, and CD HLA DR−.

The effects on the test cultures of the extracts obtained from each polymeric material were studied by comparing the optical density in the experimental series with the optical density in the control series. The optical density was recorded at 540 nm on a Sunrise analyzer (Tecan Austria GmbH, Grödig, Austria) Magellan for F50 v7.2 software (Tecan Austria GmbH, Grödig, Austria) that allowed automatic plotting of a calibration curve and determination of the concentrations of the substances under study. Then, the calculated cell relative growth intensity (RGI) expressed as a percentage was defined by determining the conditional level of the optical density.

The RGI was calculated using the following formula:

$$\mathrm{RGI}\left(\%\right)=\frac{mean\;optical\;density\;in\;the\;test\;culture}{mean\;optical\;density\;in\;the\;control\;culture}\times 100.$$

The results obtained were evaluated according to a rank scale for assessing cytotoxicity for each of the materials [51]. According to the ranking scale, the cytotoxicity levels (ranks) of materials represent toxicities as follows: 0 (100% of relative growth rate) and 1 (75–99% of relative growth rate)—nontoxic, 2 (50–74% of relative growth rate)—mild degree, 3 (25–49% of relative growth rate)—medium degree, 4 (1–24 of relative growth rate), and 5 (0% of relative growth rate)—evident toxicity.

## 3. Results and Discussion

### 3.1. Porous Structure of Polymers

**Figure 1:**
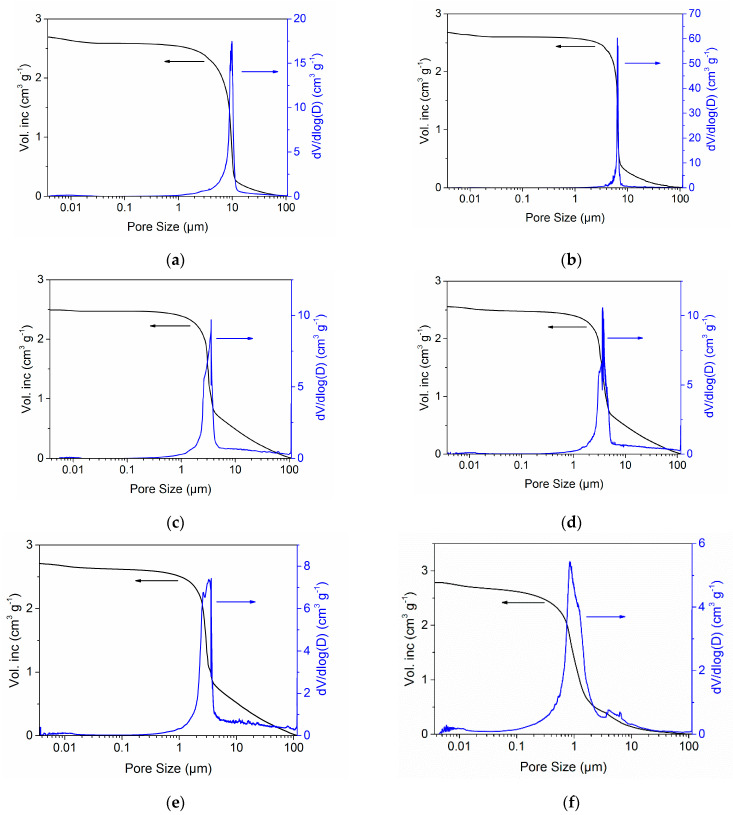
Porograms of the porous polymers obtained via polymerization of EGDMA and 1-butanol mixture, 30:70 wt% in the presence of photoinitiators (**a**–**e**), T = 25 °C and AIBN, T = 100 °C (**f**). Photoinitiators: (**a**) 36Q-amine1; (**b**) 35Q-amine1; (**c**) CQ-amine2; (**d**) PQ; and (**e**) PQ [quinone] = 0.1 wt%; and (**f**) [AIBN] = 0.1 wt%. Irradiation with Philips UHP halogen lamp (400–750 nm, 190 W), (**a**–**d**) *I* = 50 kLx and (**e**) *I* = 10 kLx.

Figure 1a–e show porograms of the porous polymers obtained from the EGDMA and 1-butanol, 30:70 wt % mixture using the studied o-quinones as photoinitiators under two modes of PPC irradiation: illuminance in the reaction zone *I* = 50 kLx (a–d) and *I* = 10 kLx (e). See Table 1 for the porous structure characteristics of the polymers (mean pore size, *D_mod_*; specific surface area, *S_sp_*; skeletal porosity, *ε_sk_*; and porosity as mercury intrusion, *ε_Hg_*). For comparison, the porogram (Figure 1e) and characteristics (Table 1) of the porous polymer obtained from the composition with the same components but under thermopolymerization in the presence of AIBN at T = 100 °C are shown.

Next follows an examination of the polymers obtained at *I* = 50 kLx. Differential curves of the dependences between the pore volume fractions and their sizes for all polymers (Figure 1a–d, blue curves) have one mode showing the mean pore size *D_mod_*. The highest value of *D_mod_* = 9.9 μm is observed for the polymer based on 36Q (36Q polymer), in which the minimum pore size is 1 μm (Figure 1a). The 35Q–based polymer (35Q polymer) has the narrowest peak of the differential curves: the minimum pore size is 3 μm, *D_mod_* = 6.4 μm (Figure 1b). The curves for the PQ– and CQ–based polymers (PQ and CQ polymers) are close to each other, and the *D_mod_* values decrease to 3.6 and 3.7 μm, respectively, compared to the 36Q and 35Q polymers where the minimum pore size is 0.8–1.0 μm (Figure 1c,d).

Examination of integral curves of the dependences of the pore volume fractions on their size (Figure 1, black curves) shows that all the polymers have large pores ≤ 100 µm in size, the fraction of large pores being greater in polymers PQ and CQ. The values of the specific surface areas *S_sp_* of the polymers obtained via photoinitiation lie in the range of 29.4 to 43.1 m^2^ g^−1^, whereas this value is 1.5–2 times higher for the AIBN polymer, reaching 60.4 m^2^ g^−1^.

The porosity as mercury intrusion, *ε_Hg_*, equivalent to the volume fraction of open pores, lies in the range 72.2–76.1% for all the polymers. Skeletal porosity, *ε_sk_*, i.e., the volume fraction of closed and open pores in the polymers, varies from 76.0 to 77.6%. The difference between these values corresponds to the volume fraction of the closed pores. It follows from Table 1 that the fraction of closed pores for polymers 36Q, PQ, and CQ is 1.1 to 2.5%, whereas the volume fraction of closed pores for polymer 35Q is maximal, amounting to 5.4%.

Thus, according to the porometry data, during polymerization of the EGDMA and 1-butanol, 30:70 wt% mixture photoinitiated by the quinones under study, in all cases, porous polymers with > 70% porosity and 100 μm maximum pore size were formed. A 75.4% (maximum) fraction of open pores with 9.9 μm (maximum) mean pore size is typical of polymer 36Q. The minimum fraction of open pores is 72.2% in polymer 35Q. The formation of large pores with sizes in the tens of microns is more characteristic of the CQ and PQ polymers.

Slowing down the formation of a porous polymer by reducing the intensity of the initiating radiation as with obtaining polymer PQ (illuminance I reduction from 50 to 10 kLx in the reaction zone) led to significant changes in the pore characteristics. Although the *D_mod_* value did not change, the mercury intrusion porosity *ε_Hg_* increased to 75.0%, and the fraction of closed pores decreased from 2.5 to 1.1% in the PQ * polymer.

Figure 1e and Table 1 show the characteristics of the polymer obtained via polymerization of the EGDMA and 1-butanol mixture, 30:70 wt%, using AIBN as a thermal initiator at T = 100 °C (AIBN polymer). It can be seen that polymerization of the mixture using thermoinitiation leads to the formation of a porous polymer with a smaller pore size compared to photoinitiation. *D_mod_* = 0.8 µm, i.e., the fraction of large pores is insignificant, which corresponds to the highest value of *S_sp_* = 60.4 m^2^ g^−1^. Furthermore, the 76.1% porosity is maximal and almost all pores in the polymer are open.

### 3.2. Scanning Electron Microscopy

**Figure 2:**
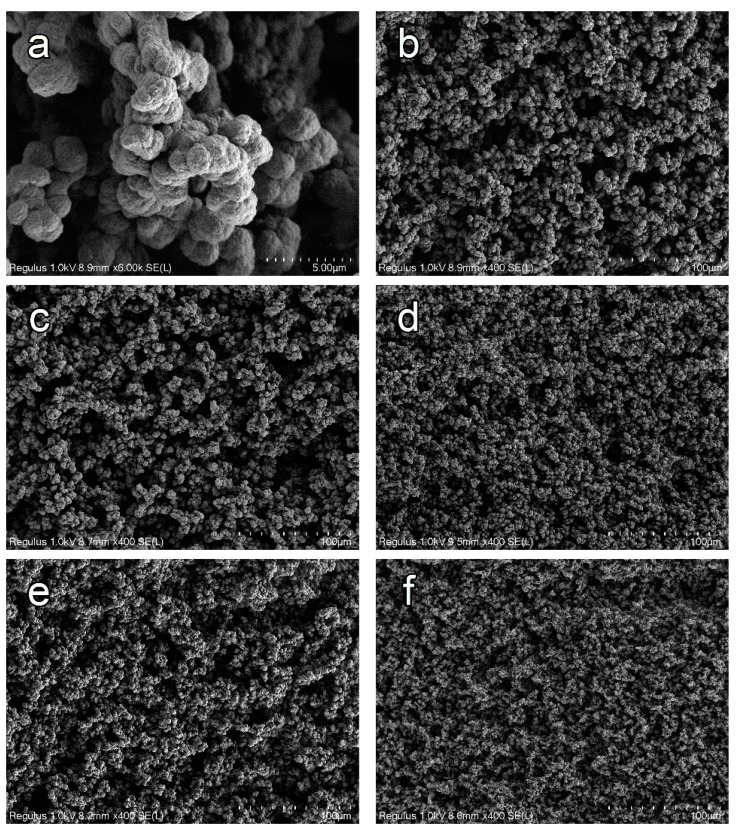
SEM images of porous polymer surfaces obtained via polymerization of an EGDMA and 1-butanol mixture, 30:70 wt%: (**a,b**) 35Q, (**c**) 36Q, (**d**) PQ, (**e**) CQ, and (**f**) AIBN.

Let us compare these results with the data obtained using scanning electron microscopy (SEM). Figure 2 shows SEM micrographs of the surfaces of the porous poly-EGDMAs formed from the EGDMA and 1-butanol composition.

Figure 2 shows SEM surface micrographs of polymers formed using 35Q, 36Q, CQ, PQ, and AIBN as initiators. It can be seen that all the resulting polymers are conglomerates of spherical particles and resemble coral. The area between the conglomerates of particles is free space making a system of interconnected pores. A typical structure of one of the porous polymers at high magnification (×6000) is shown in Figure 2a. The size of the spherical polymer particles depends on the nature of the initiator used and is about 2 μm for 35Q. Small-sized cavities are located between the polymer particles. SEM images (Figure 2b–e) at low magnification (×400) show that for all samples the spherical polymer particles form large conglomerates with large pores between them. Since the polymers were synthesized under the same conditions and from the same reaction mixture, changing only the initiating system, it is clear that pore size is determined by the nature of the initiator. It can be seen that SEM results are consistent with the mercury porometry data. Polymers 35Q and 36Q show the largest pores and spherical polymer particles. The PQ and CQ polymers with 3.6–3.7 μm pore sizes, according to mercury porometry, have smaller pores and polymer globules. A significant difference in the structure of the polymer occurs when thermoinitiated polymerization is used. The minimal sizes of pores and polymer particles are those of the AIBN sample obtained under thermopolymerization conditions.

### 3.3. Mechanical Characteristics of the Polymers

The relationship between pore architecture, pore connectivity, polymer composition, and the mechanical responses of the porous materials has been discovered and is being actively investigated [52]. The dependence of compressive strength and compression modulus on total porosity is an exponential function [53,54]. In this paper, we describe how we investigated polymers of the same composition and the same porosity (72.2–76.1%). This makes it possible to consider the influence of the pore size distribution, alone, on the Young’s modulus (*E*) and compressive breaking stress (*σ*) of the polymers.

**Figure 3:**
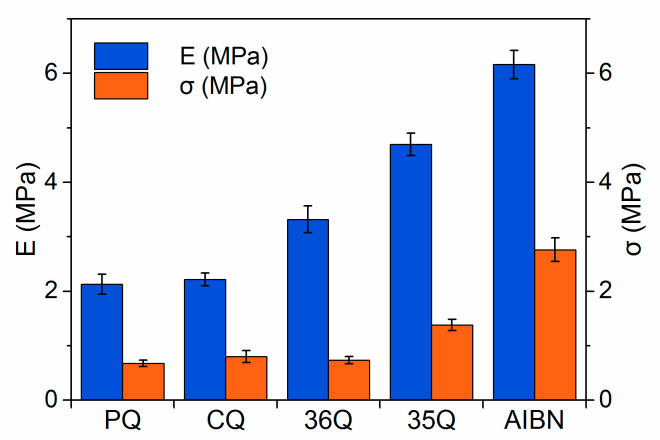
Young’s modulus (*E*) and compressive breaking stress (*σ*) values of the porous polymer monoliths obtained via polymerization of the EGDMA and 1-butanol 30:70 wt% composition in the presence of o-quinone photoinitiators (Philips UHP halogen lamp (400–750 nm, 190 W), *I* = 50 kLx, T = 25 °C) and AIBN (T = 100 °C).

The strength characteristics of the porous polymers obtained via polymerization of the EGDMA and 1-butanol composition, 30:70 wt% in the presence of all the quinone photoinitiators at *I* = 50 kLx and with AIBN are shown in Figure 3.

Among the polymers resulting from the o-quinone photoinitiators, polymer 35Q has the most promising characteristics: Young’s modulus *E* = 4.7 MPa and stress before rupture *σ* = 1.4 MPa. For the 36Q-initiated polymer, *E* = 3.3 MPa and *σ* = 0.7 MPa. On considering the PQ and CQ polymers, the value of *σ* is seen to be practically unchanged, but the Young’s modulus decreases to 2.1 and 2.2 MPa, respectively. Let us compare these results with the pore characteristics of the polymers. The values for the pore characteristics and porograms of the PQ and CQ polymers are also close (Table 1, Figure 1, porograms c and d). They are characterized by lower values of *D_mod_* compared to polymers formed using 35Q and especially 36Q. However, the strongest polymer was obtained using AIBN and had an average pore size even smaller than *D_mod_* = 0.8 μm. Obviously, the *D_mod_* value by itself is not a unique determinant of polymer strength. Another feature of polymers PQ and CQ is that their pore characteristics, compared to polymers 35Q and 36Q, show a greater contribution of large pores with ≤100 µm sizes in the structures of the polymers. Table 2 shows the pore fractions for each of the polymers in three pore size intervals: (1) 0.01 to 1.0 μm, (2) 1.0 to 12.0 μm, and (3) 12 to 100 μm. Inclusion of the maxima for the curves of pore size distribution for all the studied polymers determines the first and second intervals. Accordingly, the third interval characterizes the fraction of large pores in these polymers. It is seen that in photoinitiator-based polymers the fraction of pores smaller than 1 μm is 5% at a maximum, and 1–12 μm pores are the main contributors to the porous structure. Furthermore, the contribution of pores larger than 12 μm is also significant. In contrast, almost all pores in the AIBN polymer are smaller than 12 μm.

**Figure 4:**
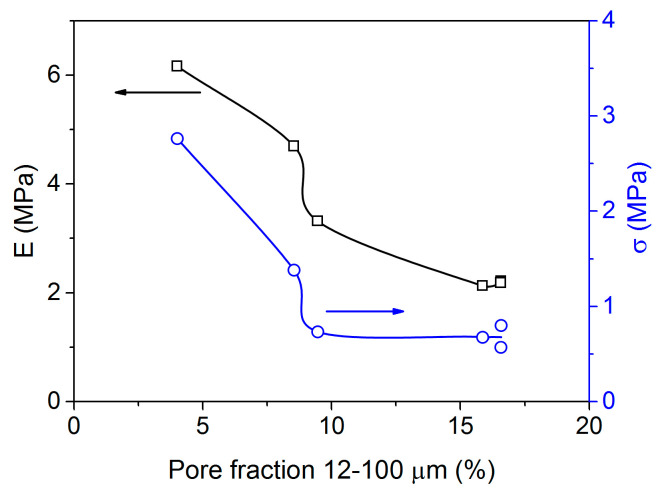
Dependence of Young’s modulus (*E*) and compressive breaking stress (*σ*) of the investigated porous polymer monoliths on *φ*_12-100_.

The fraction of 12–100 μm pores in the polymer structure *φ*_12-100_ depends on the nature of the initiator and decreases in the series: CQ > PQ > 36Q > 35Q > AIBN. Correspondingly, the *E* and *σ* values increase in reverse order (Figure 4), i.e., the larger the pores in the polymer structure, the lower its strength. This is supported by the results for polymer PQ * compared to polymer PQ. Despite the increase in the fraction of small pores to 5.64%, its strength characteristics are the same as the PQ polymer. Both the dependences (*E* = *f*(*φ*_12-100_) and *σ* = *f*(*φ*_12-100_)) have a step in the transition between the 35Q- and 36Q-based polymers. It can be assumed that the higher strength of the 35Q-based polymer is associated not only with its lower *φ*_12-100_, but also with the smaller fraction of small pores, i.e., a narrower pore size distribution in general.

### 3.4. Kinetics Studies

**Scheme 2:**
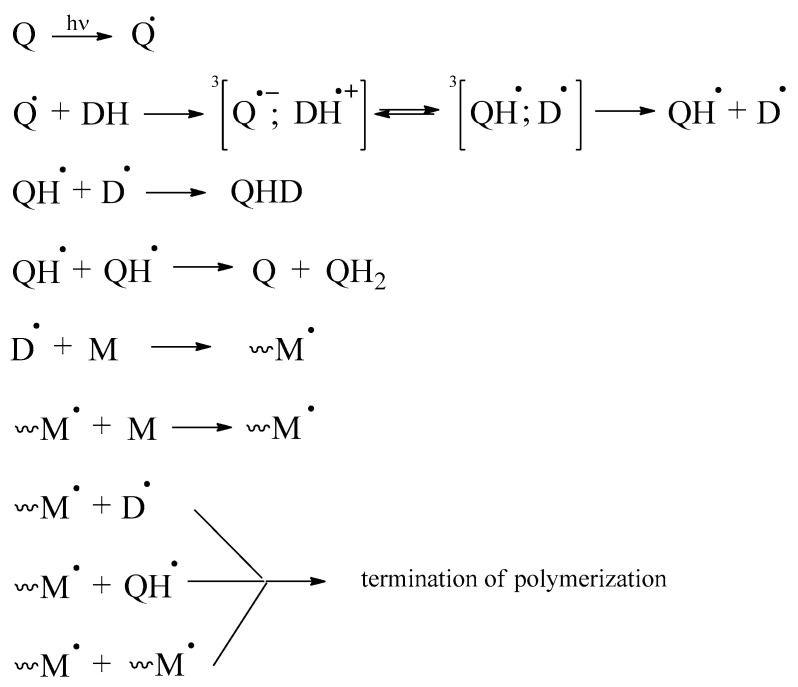
The mechanism of radical photopolymerization initiation in the presence of o-quinones.

It is important to note that under the conditions of the experiment performed to obtain polymers via photopolymerization at the same temperature T = 25 °C, the thermodynamic compatibility of the components of the polymerizing mixture (EGDMA-1-butanol—poly-EGDMA) will be the same and will not affect the process of pore formation. Therefore, different initiation efficiencies and, accordingly, different rates of polymerization are possible reasons for the influence of the nature of the initiator on the pore structure of the polymers obtained from the same starter composition. The mechanism of photopolymerization initiated by o-quinones can be described using the general Scheme 2 [23,36,55,56].

**Figure 5:**
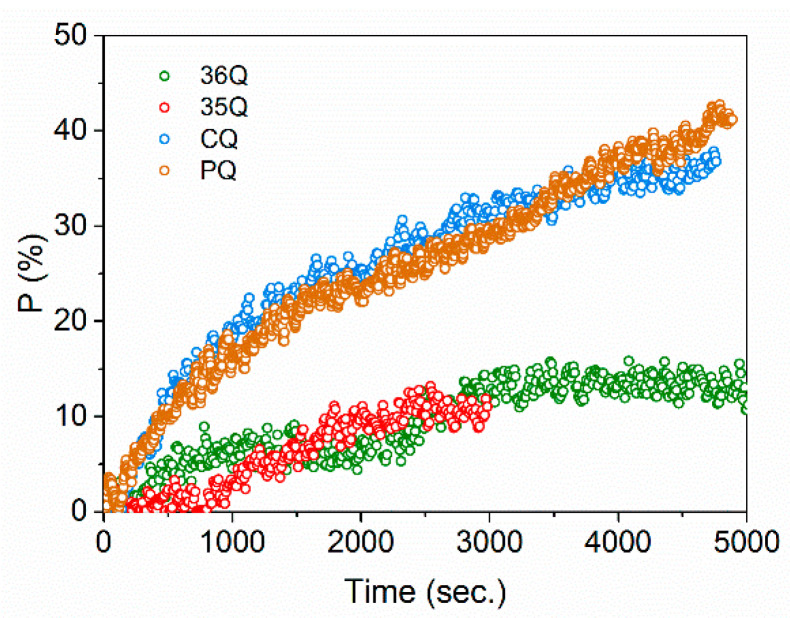
Kinetic curves for the photopolymerization of EGDMA mixed with 1-butanol, 30:70 wt% in the presence of different photoinitiators: PQ (orange curve); CQ-amine2 (blue curve); 35Q-amine1 (red curve); and 36Q-amine1 (green curve). *I* = 50 kLx.

Photoinitiation of radical polymerization in the presence of o-quinones is based on their photoreduction reaction [23,32,33,34,46,47,57]. The spectral sensitivity range of these systems is determined by the position of the quinone absorption bands. In the visible part of the spectrum, for PQ, 35Q, and 36Q solutions, two bands exist with maxima of 410 and 510 nm [58], 397 and 595 nm [23,33,57], and 410 and 598 nm [23,33,57], respectively, corresponding to the S(π→π*) and S(n→π*) electronic transitions of carbonyl groups. By absorbing visible radiation, the o-quinone molecule is excited to the S(π→π*) or S(n→π*) energy levels. From both excited states, due to internal and intercombinational conversion, the o-quinone molecule passes to the lowest excited triplet state T(n→π*). The latter is active in hydrogen stripping from H-donor molecules [57,59]. The process is similar for camphorinone, with the difference that in the visible part of the spectrum for the CQ solution there exists only one band corresponding to the S(n→π*) electronic transitions of carbonyl groups, with a maximum of 480 nm [36,37,38,39,60,61]. As a result of the interaction of the photo-excited o-quinone molecule in the lower triplet state with the hydrogen donor molecule, triplet ion-radical and radical pairs are sequentially formed, and the radical D• is generated, initiating the monomer radical polymerization. Accordingly, the higher the photoreduction rate of the o-quinone, the higher its efficiency should be as a photoinitiator of polymerization. Radical QH• can react with D• to form a phenol ester [57,62,63], and it can undergo a disproportionation reaction to produce pyrocatechin and quinone, and the D• can attach itself to a macroradical and break the polymerization chain. Inhibition of polymerization by o-quinone, itself, and by its photoreduction products reduce the photoinitiating efficiency of polymerization via o-quinones. See Figure 5 for the kinetic curves for photopolymerization of EGDMA mixed with 1-butanol, 30:70 wt% in the presence of the photoinitiating systems under study. See Table 3 for the quantitative characteristics of the photopolymerization processes.

The results above show that the studied photoinitiators fall into two groups according to their photoinitiating efficiency for the polymerization of the EGDMA and 1-butanol mixture. The photopolymerization kinetic curves are close for the use of PQ and CQ-amine2 photoinitiators, on the one hand, and for the use of 36Q-amine1 and 35Q-amine1 photoinitiators, on the other hand. For the first two, as compared to photoinitiators based on 36Q and 35Q, the maximum photopolymerization rate is 3–4 times higher, and the EGDMA limit conversion is also 2–4 times higher. The induction period when switching from PQ- to CQ- or 36Q-based systems consistently increases by 1 min or more, from 80 to 140 or 200 s, respectively, and increases dramatically to 1000 s for 35Q-amine1. The low photopolymerization rates and limit monomer conversion for PPCs with the 35Q- and 36Q-based initiating systems appear to be related to the strong negative effect of 1-butanol on the photoreduction rate of these o-quinones [58,64,65,66,67]. Furthermore, the higher inhibitory capacity of 35Q compared to 36Q leads to an additional increase in the induction period and a decrease of the *P_lim_* value [29,32]. This is confirmed by the results of experiments to determine the effective rate constant of the photoreduction reaction (*k_H_*) of quinones 35Q and 36Q in the presence of amine1 using the spectrophotometric method. EGDMA photopolymerization in an EGDMA and 1-butanol mixture is accompanied by increased turbidity of the reaction medium related to the pore formation process, thus preventing spectrophotometric determination of *k_H_* for this system. Therefore, the reaction was carried out in a mixture of DME with 1-butanol (30:70). Values of the photoreduction rate constants were determined experimentally for 35Q, *k_H_* = 1.6 × 10^−3^ s^−1^ and for 36Q, *k_H_* = 0.6 × 10^−3^ s^−1^. In other words, the photoreduction rate of 35Q and, consequently, the initiation radical generation rate are 2.5 times higher than those of 36Q. However, the photopolymerization reaction initiated by 35Q proceeds with a longer induction period, but with the same *W_max_*, as does photopolymerization in the presence of 36Q.

Comparison of the photopolymerization kinetics data for the studied PPCs with the morphology of the resulting porous polymers shows the following. The observed decrease in the proportion of large pores in the porous structure of the 36Q and 35Q polymers compared to PQ and CQ polymers is probably associated with a decrease in the rate of photopolymerization of the PPC. A slowing of the porous structure formation should increase the uniformity of both the resulting polymer and the separation of the porogen microphase. As a consequence, a more homogeneous porous structure should be formed. This conclusion is supported by the porosimetry data for polymer 35Q. Its formation occurs at a minimum rate compared to the other polymers, and it is characterized by the narrowest pore size distribution.

### 3.5. Evaluation of the Cytotoxicity of the Polymers

In assessing cytotoxicity, we were guided by the cytotoxicity level (rank) scale where values 0 and 1 correspond to nontoxic materials; value 2, to material with a mild degree of cytotoxicity; value 3, to a medium degree; and values 4–5, to expressed toxicity [51]. An increase in cytotoxicity rank a day after extraction but with no cytotoxicity remaining after 7 days usually reflects the impact of the presence of rapidly degradable components, or it may indicate insufficient material purification before the study. The occurrence of toxic effects in a seven-day-old extract, but with no similar effects in the extract when only one day old, demonstrates a more gradual yield and accumulation of toxic compounds in the experimental environment, as well as an ability for these to manifest themselves later during clinical application of such a material, potentially leading to complications in the patient.

The results obtained for all the studied materials show a 0 or 1 rank for both 1-day extracts (Table 4) and 7-day extracts (Table 5) corresponding to zero cytotoxicity. It is noted that extracts of polymeric materials based on the photoinitiators 36Q, PQ, 35Q, and CQ (photopolymerization illuminance *I* = 50 kLx) (Table 4) obtained within 1 day show a tendency to stimulate test culture proliferation. Increase of optical density was observed in the experimental series, and, as a result, there is also an increase of cell relative growth intensity (RGI), persisting to a greater or lesser extent even when the extract is diluted.

A similar trend persists in the 7-day extracts (Table 5) and is most pronounced for the PQ and CQ polymers. In addition, the 7-day extract of AIBN material also shows a tendency to stimulate test culture proliferation; this was not observed in the analysis of the 1-day extract of this material.

**Figure 6:**
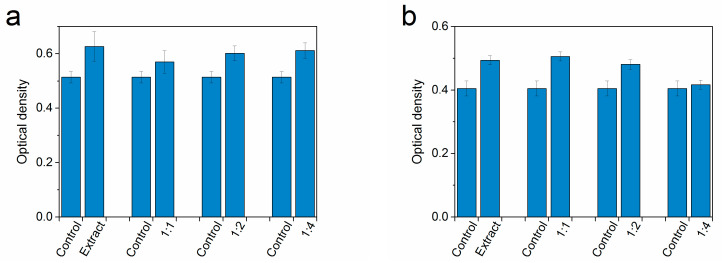
Optical density of extracts of PQ polymer samples after one day (**a**) and seven days (**b**) of extraction (MTT assay).

Thus, these results demonstrate not only the lack of cytotoxicity of all the studied materials, but also a positive effect that some materials had on the cell proliferation processes in the test cultures, this being clearly visible in the diagram for the PQ polymer presented in Figure 6. The noted trends deserve attention during subsequent work with these materials.

## 4. Conclusions

This work shows the overall possibility of using not only 3,6-di-*tret*-butyl-benzoquinone-1,2, but also using other photoinitiators of the o-quinone series (camphorquinone, 9,10-phenantrenquinone, and 3,5-di-*tret*-butyl-benzoquinone-1,2) to synthesize 2 and 4 mm-thick, porous, monolithic polymer materials. It describes an easy-to-implement method allowing the pore size and the strength characteristics of the materials to be varied, depending on the nature of the photoinitiator used. It should be noted that the application of photoinitiators sensitive to visible radiation allowed us to obtain porous polymer monoliths with mean pore sizes exceeding, by more than an order of magnitude, the pore size of samples synthesized using the classical method with AIBN as a thermal initiator. The porous polymers that we investigated exhibit zero cytotoxicity, indicating that polymer toxicity is not influenced by the changes to the photoinitiator system used. The data we have obtained on the morphology of the resulting materials, which have open systems of interconnected pores of different sizes, porosities > 70%, and zero cytotoxicity, along with the preliminary data on their positive effects on the proliferative activity of cells, demonstrate the potential value of further work with these materials for use in biomedical applications, in particular, as the basis for the development of bone-replacement materials. This applies to the greatest extent to polymers obtained using camphorquinone and 9,10-phenanthrenequinone, in which the porous structure has the largest fraction of large pores.

## Figures and Tables

**Table 1:** Properties of the porous polymers obtained via polymerization of the EGDMA and 1–butanol, 30:70 wt% mixture in the presence of o-quinone photoinitiators (Philips UHP halogen lamp (400–750 nm, 190 W), *I* = 10 (*) and 50 kLx, T = 25 °C) and with AIBN (T = 100 °C).

Polymer	*D_mod_*, μm	*ε_sk_*, %	*ε_Hg_*, %	S_sp_, m^2^ g^−1^	*ε_sk_*–*ε_Hg_*, %	*E*, MPa	*σ*, MPa
36Q	9.9	77.0	75.4	43.1	1.6	3.3	0.73
35Q	6.4	77.6	72.2	29.5	5.4	4.7	1.38
PQ	3.6	76.0	73.5	38.4	2.5	2.1	0.68
PQ *	3.6	76.1	75.0	36.3	1.1	2.2	0.57
CQ	3.7	76.2	74.5	29.4	1.7	2.2	0.80
AIBN	0.8	77.3	76.1	60.4	1.2	6.2	2.76

PQ * is a porous monolith obtained at *I* = 10 kLx.

**Table 2:** Pore size distribution in specified intervals in polymers formed from EGDMA and 1-butanol, 30:70 composition wt% obtained using different initiators.

Polymer	Pore Size/Fraction		
	0.01–1.0 μm, %	1–12 μm, %	12–100 μm, %
CQ	4.60	76.90	16.58
PQ	3.05	78.54	15.86
PQ *	5.64	76.08	16.57
36Q	3.28	85.48	9.46
35Q	1.46	89.21	8.54
AIBN	47.7	46.25	4.01

PQ * is a porous monolith obtained at *I* = 10 kLx.

**Table 3:** Kinetic characteristics of the photopolymerization of EGDMA mixed with 1-butanol, 30:70 wt% in the presence of different photoinitiators, using a Philips UHP halogen lamp (400–750 nm, 190 W, *I* = 10 and 50 kLx, T = 25 °C) in air.

Polymer	Illuminance, kLx	*T_ind_*, s	*W_max_* × 10^4^, s^−1^	*P_lim_*, %
PQ	50	80	2.7	41
CQ	50	140	2.2	35
36Q	50	200	0.7	14
35Q	50	1000	0.7	11
PQ *	10	80	1.9	29

PQ * is a porous monolith obtained at *I* = 10 kLx.

**Table 4:** Assessment of cytotoxicity of porous polymeric materials obtained using either photoinitiators (illuminance during photopolymerization *I* = 50 kLx) or AIBN. 1-day extract.

Series	Parameters	Polymer				
		36Q	PQ	35Q	CQ	AIBN
Control (n = 8)	OD	0.455 ± 0.08	0.524 ± 0.021	0.454 ± 0.029	0.537 ± 0.014	0.553 ± 0.015
	RGI, %	100	100	100	100	100
	Cytotoxicity level	0	0	0	0	0
Extract (n = 8)	OD	0.559 ± 0.018	0.627 ± 0.054	0.584 ± 0.022	0.679 ± 0.041	0.457 ± 0.021
	RGI, %	122	119	129	126	83
	Cytotoxicity level	0	0	0	0	1
Extract 1:1 (n = 8)	OD	0.496 ± 0.012	0.57 ± 0.041	0.517 ± 0.017	0.615 ± 0.023	0.505 ± 0.026
	RGI, %	109	108	114	115	91
	Cytotoxicity level	0	0	0	0	1
Extract 1:2 (n = 8)	OD	0.485 ± 0.016	0.602 ± 0.027	0.585 ± 0.013	0.674 ± 0.018	0.422 ± 0.014
	RGI, %	106	115	129	126	76
	Cytotoxicity level	0	0	0	0	1
Extract 1:4 (n = 8)	OD	0.507 ± 0.018	0.612 ± 0.028	0.533 ± 0.027	0.722 ± 0.022	0.436 ± 0.013
	RGI, %	111	117	117	134	79
	Cytotoxicity level	0	0	0	0	1

**Table 5:** Assessment of cytotoxicity of porous polymeric materials obtained using either photoinitiators (illuminance during photopolymerization *I* = 50 kLx) or AIBN. 7-day extract.

Series	Parameters	Polymer				
		36Q	PQ	35Q	CQ	AIBN
Control (n = 8)	OD	0.372 ± 0.023	0.405 ± 0.021	0.417 ± 0.016	0.386 ± 0.014	0.370 ± 0.013
	RGI, %	100	100	100	100	100
	Cytotoxicity level	0	0	0	0	0
Extract (n = 8)	OD	0.380 ± 0.01	0.494 ± 0.014	0.427 ± 0.011	0.494 ± 0.023	0.429 ± 0.014
	RGI, %	102	121	102	128	116
	Cytotoxicity level	0	0	0	0	0
Extract 1:1 (n = 8)	OD	0.385 ± 0.009	0.506 ± 0.014	0.416 ± 0.012	0.483 ± 0.024	0.467 ± 0.013
	RGI, %	103	125	99	125	126
	Cytotoxicity level	0	0	1	0	0
Extract 1:2 (n = 8)	OD	0.420 ± 0.013	0.481 ± 0.015	0.429 ± 0.008	0.422 ± 0.010	0.439 ± 0.009
	RGI, %	112	119	103	109	119
	Cytotoxicity level	0	0	0	0	0
Extract 1:4 (n = 8)	OD	0.439 ± 0.013	0.417 ± 0.014	0.417 ± 0.007	0.367 ± 0.023	0.475 ± 0.021
	RGI, %	118	103	100	95	128
	Cytotoxicity level	0	0	0	1	0

## Data Availability

The raw/processed data required to reproduce these findings cannot be shared at this time as the data also form part of an ongoing study.

## Sample Availability

Samples of the compounds are available from the authors.

## Dedication

Dedicated to the memory of corresponding member of the RAS, Vladimir Cherkasov.
